# Late Presentation of an Iatrogenic Pseudoaneurysm of the Profunda Femoris Artery following Intramedullary Nailing

**DOI:** 10.1155/2018/8270256

**Published:** 2018-09-23

**Authors:** Kira Vande Voorde, Jan Dauwe, Jan Van Oost

**Affiliations:** ^1^Department of Orthopaedic and Trauma Surgery, AZ Delta Hospital, Belgium; ^2^Department of Orthopaedic Surgery, University Hospitals Leuven, Leuven, Belgium

## Abstract

**Introduction:**

Hip fractures are one of the most common osteoporotic fractures, and the incidence is expected to increase in the future. Vascular injury of the femoral vessels, although uncommon, is an intermittently reported complication in the treatment of proximal femoral fractures. This may be iatrogenic or less frequently as a result of the fracture itself. The profunda femoris artery is most commonly involved, probably because of its close relationship to the femur in the subtrochanteric region.

**Case Presentation:**

We report a well-documented case of pseudoaneurysm of the profunda femoris artery after intramedullary nailing of an intertrochanteric femoral fracture. Arterial damage was due to overpenetration when drilling the distal locking hole. Because of the late presentation, pressure on the medial femoral diaphysis caused severe cortical scalloping. This resulted in an obvious radiographic image rarely reported before.

**Conclusion:**

This case report illustrates the uncommon complication of pseudoaneurysm after intramedullary hip nailing. Because of the risk of potentially limb- and life-threatening complications, we advise careful drilling and placement of the distal locking screw. Excessive screw length should be avoided. The injured limb should be returned to the neutral position and lower-limb traction should be reduced before drilling the distal locking hole.

## 1. Introduction

Hip fractures are one of the most common osteoporotic fractures, and the incidence is expected to increase in the future [1, 2]. Different treatment options for internal fixation in trochanteric fractures, such as intramedullary nailing, have been abundantly described in the literature [2].

Vascular injury of the femoral vessels in intertrochanteric fractures is a rare complication in the treatment of proximal femoral fractures [3]. This may be iatrogenic or less frequently a result of the fracture itself, caused by vessel damage secondary to sharp bony fragments [4]. Iatrogenic vascular injury can be caused by either the use of retractors [5], drilling the distal locking hole, or protrusion of the distal locking screw through the medial femoral cortex [3, 6–8]. Profunda femoris artery (PFA) damage, injury of the superficial femoral artery (SFA), or their perforating branches can cause acute hemorrhage, pseudoaneurysms, or compartment syndrome and may require vascular surgery [4, 9]. Pseudoaneurysm and intramuscular hematoma are potentially limb- and life-threatening [10].

We report a well-documented case of an uncommon complication resulting in pseudoaneurysm after intramedullary nailing of an intertrochanteric femoral fracture. Because of the late presentation, progressive formation of the pseudoaneurysm resulted in pressure on the medial femoral diaphysis with scalloping and increased risk of fracture. This resulted in an outspoken radiographic image rarely reported before.

## 2. Case Presentation

**Figure 1:**
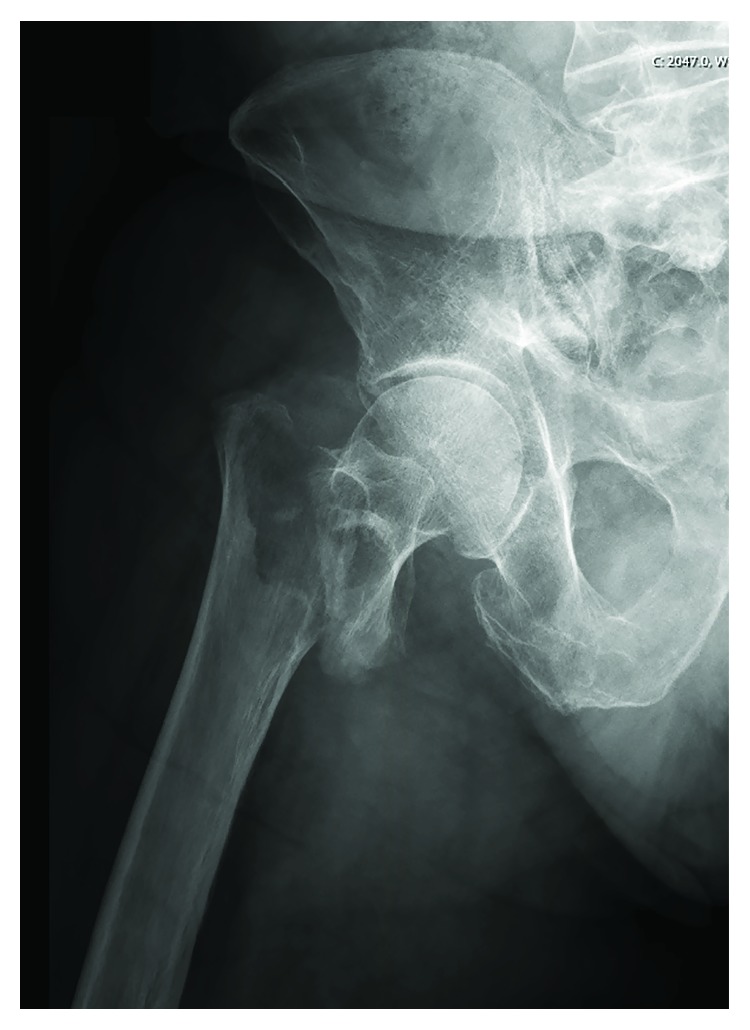
Plain X-ray of the painful hip and pelvis diagnosed a comminuted intertrochanteric hip fracture (grade 31 A2 according to the AO classification).

A 78-year old Caucasian male patient, without relevant medical history and in good health, presented to the emergency department with severe pain in the right hip after a high energy trauma due to a fall. On clinical examination, the right leg was shortened and externally rotated. There was no neurovascular deficit in the ipsilateral limb. A comminuted intertrochanteric hip fracture was diagnosed on a plain X-ray of the painful hip and pelvis (grade 31-A2 according to the AO classification) (Figure 1). Intravenous pain medication was administered at the emergency department, and the patient was transferred to the operating room five hours after admission.

Closed reduction and internal fixation with a 170 mm 125° intramedullary nail (proximal femoral nail antirotation (PFNA; Synthes®), 11 mm diameter) were performed. The patient was placed supine on a fracture table with traction and the hip in adduction and internal rotation. The nail could be introduced without any particular difficulty. A 115 mm blade and a 38 mm distal locking screw were inserted with the use of the aiming arm. For distal locking, a drill sleeve, a protection sleeve, and a 4.2 mm calibrated drill bit (340 mm) were used. Drilling was guided, however not guarded. There were no intraoperative or immediate postoperative complications. Postoperative radiographs were satisfactory. Postoperatively, three weeks nonweight bearing were instructed because of the high energy impact of the trauma. Also, low molecular weight heparin (Enoxaparin 40, 1 subcutaneous injection per day) was administered for six weeks.

**Figure 2:**
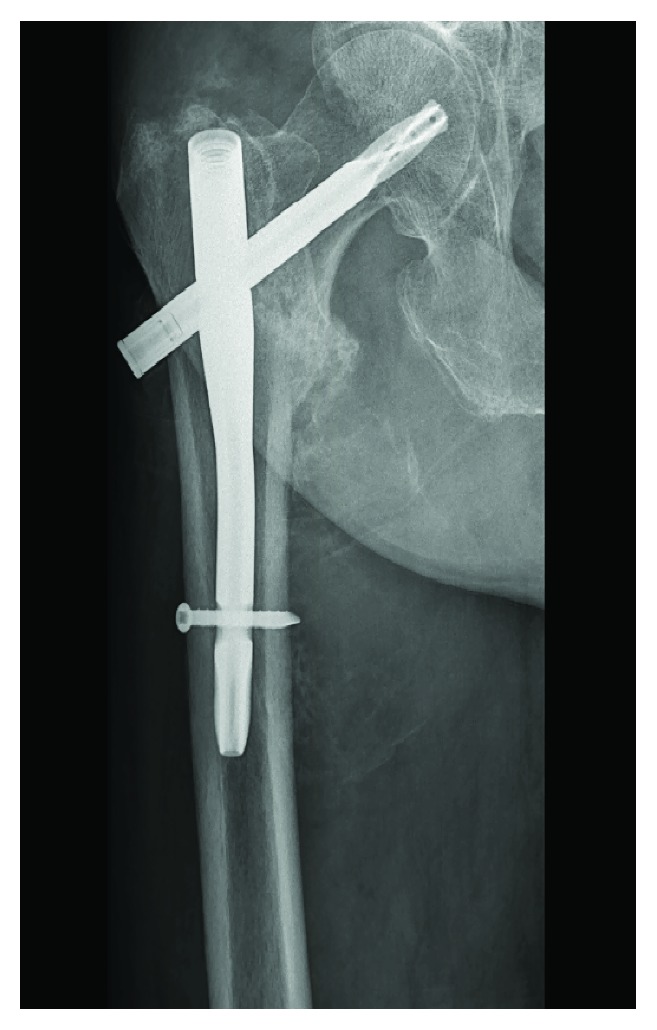
Plain X-ray of the right hip six weeks postoperatively showed a good position of the intramedullary nail.

On clinical and radiographic checkup six weeks after surgery, no particular difficulties were noticed. The patient was able to walk with one crutch, there was no obvious swelling of the limb, and radiographs showed a good position of the intramedullary nail (Figure 2).

**Figure 3:**
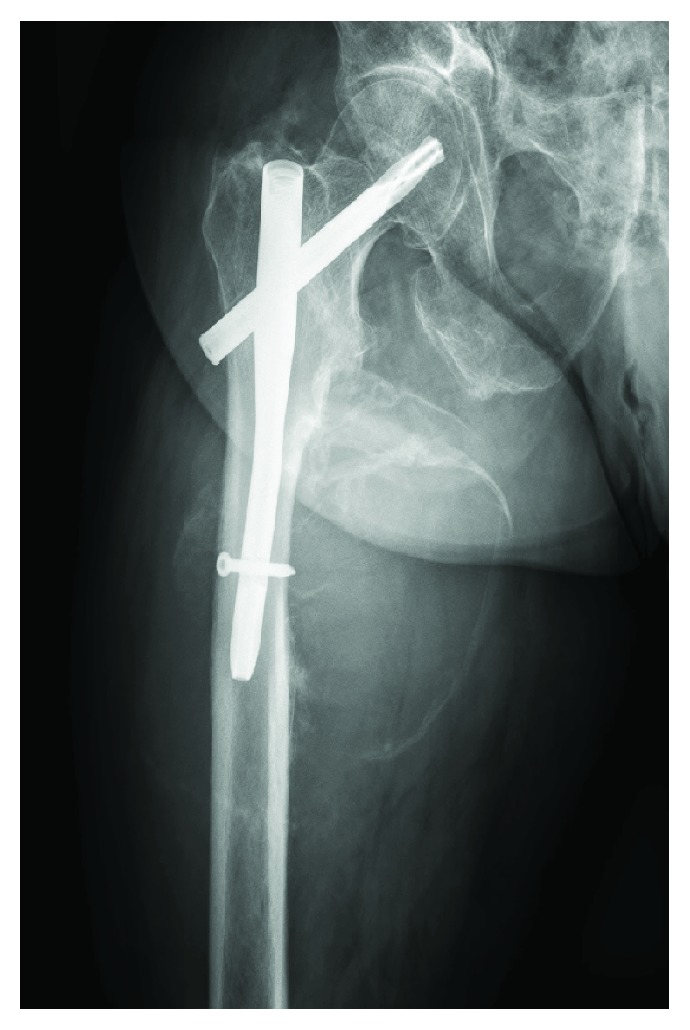
Plain X-rays eight months postoperatively showed the presence of a medial mass centred over the distal locking screw with calcification of the peripheral borders, suggestive for an (infected) hematoma. Compression and severe osteolysis of the medial border of the femur were remarked.

**Figure 4:**
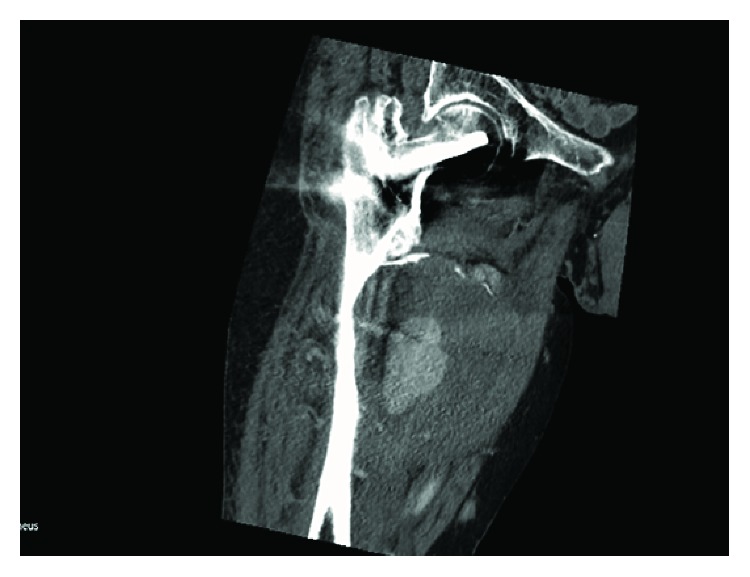
Contrast-enhanced computed tomography (CT) with IV contrast showed an active extravasation in the hematoma which concluded to the diagnosis of pseudoaneurysm. Severe scalloping of the medial femoral cortex was confirmed.

Eight months postoperatively, the patient presented to polyclinical consultation because of a progressive swelling of the right thigh. There was no recent trauma, episode of fever, or illness. Clinical examination revealed a nontender diffuse swelling over the proximal part of the right thigh without well-defined borders, redness, or fluctuation. Peripheral pulses of the lower limbs were palpable, although stronger in the left limb, and capillary refill was normal. Mobilisation of the hip was painless. Ultrasound revealed a calcified old muscular hematoma localised medial to the femoral diaphysis. Additional X-rays confirmed the presence of a medial mass centred over the distal locking screw with calcification of the peripheral borders, suggestive for an (infected) hematoma. Compression and severe osteolysis of the medial border of the femur were seen (Figure 3). Contrast-enhanced computed tomography (CT) was performed, showing an active extravasation in the hematoma which led to the diagnosis of pseudoaneurysm (Figure 4). Laboratory tests showed a haemoglobin value of 11 g/dL and an elevated CRP (42 mg/L). Other laboratory parameters were within normal range. On retrospective analysis of the X-rays six weeks postoperatively, the additional mass could have been already noticed. However, because of the very subtle signs, it was misdiagnosed.

The patient was referred to the department of vascular surgery for open drainage. Preoperative findings showed a PFA lesion facing the distal locking screw which was managed by direct arterial suture. The screw was not visible and therefore left in place. No weight-bearing limits were made within the context of the scalloping of the medial femoral cortex because of patient's compliance and a good consolidation of the fracture. The patient was followed up regularly, and further evolution was uneventful.

## 3. Discussion

Hip fractures are one of the most common osteoporotic fractures, and the incidence is expected to increase in the future [1, 2]. Different treatment options for internal fixation of intertrochanteric femoral fractures have been described in the literature. Intramedullary nailing (i.e., PFNA; Synthes® and Gamma Nail; Stryker®) and extramedullary fixation (dynamic hip screw (DHS); Synthes®) are the two most often performed procedures for treatment. A meta-analysis of current evidence indicates that PFNA, because of its minimal invasiveness, may be a better choice than dynamic hip screw fixation in the treatment of intertrochanteric hip fractures. Therefore, PFNA is recommended in the treatment of elderly patients with an intertrochanteric hip fracture [11].

Vascular injury following hip fractures is a rare (incidence of 0.2% [12]) but serious complication that has been intermittently reported in the literature. Arterial vessel damage may be caused by the fracture itself or more commonly as a consequence of intertrochanteric fracture repair [7]. Avulsion of the lesser trochanter leads to an increased risk of femoral vessel damage caused by a displaced bone spike [4]. Iatrogenic vascular injury can be caused either by the use of retractors, by drilling the distal locking hole, or by protrusion of the distal locking screw through the medial femoral cortex [3, 6–8]. Pseudoaneurysms or false aneurysms are caused by partial arterial vessel damage with subsequent formation of a hematoma that is in contact with the arterial lumen. The hematoma is contained by the adventitia or surrounding soft tissues. Damage to the profunda femoris artery (PFA) or its perforating branches is regularly reported, probably because of their close relationship to the femur in the subtrochanteric region [12]. The injury of the superficial femoral artery (SFA) or its branches are less frequent [3].

In the present case, the distal locking screw was not visible during open exploration and we concluded that the pseudoaneurysm was not the result of a vascular friction lesion. We suggest that the PFA damage was iatrogenic due to overpenetration when drilling the distal locking hole, since drilling was guided but not guarded. Subsequent formation and progressive growth of pseudoaneurysm resulted in pressure on the medial femoral diaphysis with scalloping of the cortex and an increased risk of refracture (Figure 3). Because of the late presentation, this resulted in an obvious radiographic image, which is rarely seen. However, it is known that the time lag between injury and diagnosis of pseudoaneurysms may vary from hours to years. When damage is caused by a bony fragment or iatrogenic during surgery by overpenetration by a drill bit, retractor, or screw, the onset of the symptoms is more acute. A vascular friction lesion due to the distal locking screw protruding the medial femoral cortex mostly results in a delayed onset of symptoms [12]. Postoperative persistent hip pain, progressive thigh swelling, and anaemia should arouse suspicion but are nonspecific clinical features, not uncommon after hip surgery, and therefore sometimes misdiagnosed [7]. Retrospectively, in our patient, clinical and radiographic suspicions should have been aroused at six weeks of follow-up, but the subtle calcified hematoma on X-ray was not noticed.

Because of the risk of potentially limb- and life-threatening complications like bleeding, infection, compression of adjacent structures, and compartment syndrome [7], accurate diagnosis and assessment are crucial. A high index of suspicion supported by additional radiological imaging such as (Doppler) ultrasound, computed tomography, and angiography are necessary to reach the diagnosis [13].

To prevent the occurrence of pseudoaneurysm or other vascular injuries after hip fracture surgery, the relationship between distal locking screws and femoral arteries and the safe region for distal screw placement in closed hip nailing was studied (Asian population). Han et al. [9] and Gong et al. [12] concluded that the PFA was closest to the distal locking screw of the short nails (170 mm PFNA, 200 mm PFNA-II, and 180 mm ITST) and therefore short nails were most at risk to cause PFA damage. Short nails were relatively more distant from the SFA, which was located approximately 20–30 mm from the femoral far cortex [9] and posteriorly to the long nails (300 mm PFNA-II and 300 mm ITST). Consequently, long nails are less likely to cause injury. However, positioning of the affected limb in adduction and internal rotation during surgery may alter the position of the vessels in the thigh and decrease the distance between the nail and the femoral arteries, especially the SFA. This was also described by Grimaldi et al. who stated that patient installation on the traction table impacts the risk of femoral vessel damage in several ways, especially SFA damage. Besides an altered position of the vessels due to adduction and internal rotation, lower-limb traction also reduces mobility of the vessels and prevents them to move out of the way of lesion-causing agents. Thirdly, the soft tissue of the thigh is compressed between the femur and perineal support of the traction table [3]. Collected data on the perforating arteries by Han et al. showed that these arteries were located within the insertion region of the distal locking screw of short nails and within the insertion region of the third and fourth distal screw of a dynamic hip screw [9]. In contrast, Gong et al. did not find perforating arteries in the risky or hazardous regions [12]. Vascular injury after DHS fixation is also reported in the literature, although less frequently. A possible explanation could be that DHS fixation needs less adduction of the limb during the procedure compared with closed hip nailing [14].

In order to minimize the risk of iatrogenic vascular injury, some recommendations are defined in the literature. After placement of the blade and before drilling an interlocking hole, the position of the injured limb should be returned to a neutral position to increase the distance between the femur and femoral vessels and lower-limb traction should be reduced to increase vessel mobility [3, 12]. Furthermore, careful drilling of the interlocking hole, the use of a guarded drill bit, careful tapping, appropriate screw length, and careful placement of retractors and clamps during surgery are mandatory [7, 12]. Screws should be long enough to penetrate the far cortex, but not longer.

In conclusion, this case report illustrates the rare but serious complication of pseudoaneurysm after intramedullary hip nailing. Because of the late presentation, pressure on the medial femoral diaphysis caused severe cortical scalloping. This resulted in an obvious radiographic image rarely reported before. We believe that orthopaedic trauma surgeons should be aware of vascular injury associated with intertrochanteric femoral fractures, although uncommon. Avulsion of the lesser trochanter is a predisposing factor for femoral vessel damage caused by a displaced bony spike. Iatrogenic vascular injury is more common and therefore we advise careful drilling and placement of the distal locking screw with an appropriate screw length. Furthermore, the injured limb should be returned to neutral position and lower-limb traction should be reduced before drilling the distal locking hole in order to prevent postoperative complications.

## References

[1] Kanis J. A., Black D., Cooper C. (2002). A new approach to the development of assessment guidelines for osteoporosis. *Osteoporosis International*.

[2] Dauwe J., Verhulst K., Grechenig P. (2017). The 25 highest cited papers in trochanteric fractures. A systematic review. *BioMedicine and Surgery*.

[3] Grimaldi M., Courvoisier A., Tonetti J., Vouaillat H., Merloz P. (2009). Superficial femoral artery injury resulting from intertrochanteric hip fracture fixation by a locked intramedullary nail. *Orthopaedics & Traumatology, Surgery & Research*.

[4] Potenza V., Saputo U., Catellani F., Farsetti P., Caterini R. (2016). Laceration of a branch of the profunda femoris artery caused by a spike of the displaced lesser trochanter in an inter-trochanteric femoral fracture. A case report. *International Journal of Surgery Case Reports*.

[5] Zhang B. F., Cong Y. X., Wang P. F., Huang H., Wang H., Zhuang Y. (2018). Deep femoral artery branch pseudoaneurysm formation and injury after hip fracture surgery: a case series and a literature review. *Medicine*.

[6] Lazarides M. K., Arvanitis D. P., Dayantas J. N. (1991). Latrogenic arterial trauma associated with hip joint surgery: an overview. *European Journal of Vascular Surgery*.

[7] Singh S., Arora S., Thora A., Mohan R., Sural S., Dhal A. (2013). Pseudoaneurysm of profunda femoris artery following dynamic hip screw fixation for intertrochanteric femoral fracture. *Chinese Journal of Traumatology*.

[8] Yang K. H., Park H. W., Park S. J. (2002). Pseudoaneurysm of the superficial femoral artery after closed hip nailing with a gamma nail: report of a case. *Journal of Orthopaedic Trauma*.

[9] Han C. D., Lee Y. H., Yang K. H. (2013). Relationship between distal screws and femoral arteries in closed hip nailing on computed tomography angiography. *Archives of Orthopaedic and Trauma Surgery*.

[10] Chan W. S.-W., Kong S.-W., Sun K.-W., Tsang P.-K., Chow H.-L. (2010). Pseudoaneurysm and intramuscular haematoma after dynamic hip screw fixation for intertrochanteric femoral fracture: a case report. *Journal of Orthopaedic Surgery*.

[11] Zhang K., Zhang S., Yang J. (2014). Proximal femoral nail vs. dynamic hip screw in treatment of intertrochanteric fractures: a meta-analysis. *Medical Science Monitor*.

[12] Gong J., Liu P., Cai M. (2017). Imaging evaluation of the safe region for distal locking screw of proximal femoral nail anti-rotation in patients with proximal femoral fracture. *Medical Science Monitor*.

[13] Roy K. D., Aggarwal R. A., Purohit S., Bandagi G., Marathe N. (2016). Iatrogenic pseudo-aneurysm of profunda femoris artery following fixation of intertrochanteric femur fracture – a case report and review of literature. *Journal of Clinical and Diagnostic Research*.

[14] Yang K. H., Yoon C. S., Park H. W., Won J. H., Park S. J. (2004). Position of the superficial femoral artery in closed hip nailing. *Archives of Orthopaedic and Trauma Surgery*.

